# Evaluation of the GenoScreen Deeplex Myc-TB workflow for drug-resistant tuberculosis testing in South Africa

**DOI:** 10.3389/fpubh.2026.1834699

**Published:** 2026-07-07

**Authors:** Jennifer Williams, Janré Steyn, Melanie Grobbelaar, Fahd Naufal, Yonas Ghebrekristos, Nabila Ismail, Jason Limberis, Christoffel Opperman, Sarishna Singh, Rouxjeane Venter, Brendon C. Mann, Felicia B. Hunter, Alina Nalyvayko, Grant Theron, Robin M. Warren, John Z. Metcalfe

**Affiliations:** 1 Division of Molecular Biology and Human Genetics, SAMRC Centre for TB Research, DSI-NRF Centre of Excellence for Biomedical TB Research, Department of Biomedical Sciences, Faculty of Medicine and Health Sciences, Stellenbosch University, Cape Town, South Africa; 2 Division of Pulmonary and Critical Care Medicine, San Francisco General Hospital and Trauma Center, University of California, San Francisco, CA, United States; 3 National Health Laboratory Service, Green Point Tuberculosis Laboratory, Cape Town, South Africa; 4 Division of Medical Microbiology, Department of Pathology, University of Cape Town, Cape Town, South Africa

**Keywords:** Deeplex Myc-TB, drug-resistant tuberculosis, targeted next-generation sequencing, tuberculosis, workflow optimisation

## Abstract

Tuberculosis (TB) remains a leading cause of death globally, particularly in low- and middle-income countries, where healthcare systems are under-resourced and the burden of drug-resistant disease is highest. We assessed the feasibility of future integration of the GenoScreen Deeplex Myc-TB assay - a targeted next-generation sequencing (tNGS) assay for *Mycobacterium tuberculosis* detection and drug-resistance profiling within a national reference laboratory in South Africa. Rather than reporting routine implementation with patient testing in parallel to standard care, this study evaluated pre-implementation readiness through workflow optimisation, infrastructure preparation, staff training, and troubleshooting across the sequencing pathway. The main challenges identified were DNA extraction from complex specimens, targeted PCR quality control, manual library preparation, sequencing consistency, proprietary analysis software, and procurement and cost constraints. Overall, these findings show that the Deeplex Myc-TB assay has potential to complement routine diagnostic testing in high-burden settings, but that sustained adoption will require appropriate extraction methods, additional local quality control measures, reliable procurement pathways, recognised reporting, and longer-term investment in laboratory and data systems.

## 1 Introduction

Tuberculosis (TB) remains a leading cause of death globally, particularly in low- and middle-income countries (LMICs) where healthcare systems are under-resourced, and the disease burden is highest. Drug-resistant TB (DR-TB), including multidrug-resistant (MDR-TB) and extensively drug-resistant TB (XDR-TB), further complicate treatment and control efforts, increasing mortality and straining public health systems. A major barrier to effective management is the delay associated with phenotypic drug susceptibility testing (pDST) (1). While World Health Organization (WHO) endorsed molecular TB diagnostic assays have improved speed and sensitivity compared with culture-based methods, they are limited to a small set of resistance-associated mutations (2).

The targeted next-generation sequencing (tNGS) assay by GenoScreen (Lille, France), Deeplex^®^ Myc-TB- provides rapid, comprehensive drug resistance profiles for up to 13 anti-TB agents with kit-based workflows and automated data interpretation, without the need for culture and high-level biosafety controls (3). The assay’s capacity to identify minority variants at frequencies as low as 3% enhances detection of heteroresistance, which is important for identifying mixed or emerging drug-resistant populations (4). While diagnostic accuracy data from LMIC settings for these technologies have been promising, they may have been evaluated under conditions that do not reflect routine use, such as highly experienced laboratories and intensive pre-study training conducted by developers and experienced manufacturers (5–7). Data on implementation of these technologies in pragmatic settings such as routine national health laboratories is limited (8).

To address this gap, we undertook an operational assessment of the GenoScreen Deeplex Myc-TB assay’s workflow in a large National Health Laboratory Service (NHLS) reference laboratory serving the Western Cape Province of South Africa - a region with high TB and DR-TB incidence (9). The aim was to identify the practical requirements and constraints for future integration of the assay into routine drug-resistant TB diagnostic workflows, with particular attention to laboratory setup, workflow optimisation, quality control, data interpretation, procurement, and sustainability.

## 2 Workflow framework and evaluation setting

### 2.1 Evaluation setting and intended use

The Deeplex Myc-TB assay was evaluated at the NHLS, Green Point, Cape Town, South Africa. This centralised facility serves as the primary reference laboratory for the Western Cape Province. The existing diagnostic workflow followed the national TB diagnostic algorithm (10) further described in Supplementary file 1. After specimens were processed via the routine workflow, we collected an aliquot of MGIT culture or decontaminated sputum sediment to process and use for downstream tNGS (11), with measures in place to prevent contamination or disruption of routine procedures (Supplementary Figure 1). All tNGS data processing was conducted independently of routine diagnostics, and results were excluded from clinical decision-making.

### 2.2 Preparation phase

Evaluation of the Deeplex Myc-TB assay required targeted infrastructure upgrades to meet sequencing and biosafety standards. Although existing molecular diagnostic capacity included PCR systems and biosafety-compliant specimen handling areas, sequencing required additional specialised equipment, workspace reconfiguration (Supplementary Figure 2), and enhanced contamination control measures (Supplementary file 1).

#### 2.2.1 Ethics approval and regulatory authorisation

The study received ethical approval from the Stellenbosch University Health Research Ethics Committee (N21/09/093; Project ID: 22873) and the University of California, San Francisco Institutional Review Board (IRB #20–33,172). Additional approvals were obtained from the National Health Laboratory Service (NHLS-AARMS, PR2120530), the Western Cape Department of Health (WC-DoH, WC_202112_021), and City of Cape Town Health (CCT-DoH, 9,803). All specimen handling, sequencing procedures and data management were conducted in accordance with applicable national ethical and data governance requirements.

#### 2.2.2 Capacity development, training, and workflow discipline

Capacity building represented a critical component of the implementation process. Successful adoption of the Deeplex Myc-TB workflow required extensive training in specimen handling, DNA extraction, PCR setup, library preparation, sequencing, and data interpretation. Laboratory personnel were trained through structured sessions with application specialists, followed by hands-on practice and supervised sessions as the workflow was established. Failed sequencing runs were followed by repetition of the workflow under supervision to identify operator-related errors and opportunities for optimisation, further described in Supplementary file 1.

### 2.3 Workflow domains assessed

We focused on the main workflow domains expected to determine future routine adoption: DNA extraction, targeted PCR, library preparation and sequencing, data analysis and reporting, and procurement and cost.

#### 2.3.1 DNA extraction

During the workflow evaluation, several limitations were identified. These include a largely manual workflow that introduced operator-dependent variability, increased hands-on time, and constrained scalability. Given the cost and maintenance requirements of automation platforms, we focused on optimising and strengthening a manual DNA extraction workflow applicable to routine laboratories in LMIC settings.

Optimisation of DNA extraction proved pivotal to assay success. The manufacturer’s recommended manual extraction method (12) did not yield DNA of sufficient quantity or quality under our laboratory conditions. To address this, we optimised a modified InstaGene/FastPrep protocol incorporating an AMPure XP bead purification step, which yielded consistent, high-purity DNA extracts suitable for downstream amplification (13). Sequencing performance remained robust even with low genomic DNA input for the Deeplex Myc-TB PCR step, with successful amplification observed from a 0.95 ng total DNA input (corresponding to a smear-positive, 1 + smear grade specimen), far below the manufacturer’s nominal recommendation of 900 ng (12). The maximum input evaluated in this study was 290.7 ng. Conversely, excessive DNA input from processed sputum sediments actually inhibited Deeplex PCR in our workflow, consistent with previous reports indicating that high template concentrations or inhibitor-rich respiratory specimens, including processed sputum and MGIT material, can adversely affect PCR amplification (14, 15).

Despite optimisations, several challenges persisted. DNA extraction from sputum sediment remained technically challenging because of the complex matrix and the presence of PCR inhibitors (16). Residual inhibitors such as polysaccharides and salts occasionally reduced amplification efficiency. In addition, import-dependent procurement, variable lead times, and delays in reagent delivery affected workflow continuity. In paucibacillary (smear-negative) specimens, sequencing performance remained suboptimal and declined sharply below a total DNA input threshold of ~0.47 ng, consistent with previous reports of poor DNA recovery from low-bacillary-load specimens (5, 17, 18). While our optimisation focused on manual extraction, automated extraction platforms may improve reproducibility and throughput in higher-capacity settings, as demonstrated by Colman *et al.* (2025) using the Maxwell platform (Promega, WI, USA) (5).

#### 2.3.2 Deeplex Myc-TB targeted PCR

Amplicon generation, like DNA extraction, required optimisation beyond the manufacturer’s instructions. During training and early feasibility testing, the workflow was followed as recommended, but minor adjustments were required. Notably, the internal control failed consecutively for kits from the same batch, despite successful amplification of the positive control and specimen-derived DNA extracts. To resolve this, we performed in-house troubleshooting and diluted the internal control with 448.5 μL instead of the recommended 897 μL of nuclease-free water. In addition, the internal control was reconstituted using nuclease-free water preheated to 90 °C to promote complete dissolution and reduce the risk of false internal control failure. This adjustment was not described in the manufacturer’s manual, was continued irrespective of batch number and highlights the importance of lot tracking, control monitoring, and predefined troubleshooting during tNGS implementation.

The manufacturer’s guidelines on assessing amplification success prior to library preparation relied primarily on post-cleanup amplicon quantification, measured using a Qubit 4 fluorometer or SYBR Green quantification on a microplate reader, with interpretation based on concentration thresholds (12). However, under our study conditions Qubit quantification alone proved insufficient because total DNA concentration did not consistently reflect multiplex completeness or confirm the presence of all expected targets. Microplate-based quantification would have required additional equipment and similarly measured only total DNA concentration.

We therefore added gel electrophoresis as an additional quality control step during workflow optimisation, to confirm successful amplification prior to sequencing (Supplementary Figure 3). This gel-based verification proved both effective and affordable.

#### 2.3.3 Library preparation and targeted NGS

Library preparation was facilitated by the assay being supplied in an integrated, end-to-end kit format, with all core reagents included (i.e., not assembled from multiple suppliers) and stable for several months, providing logistical consistency throughout the testing period. During the early stages of this study, a combined Deeplex Myc-TB/Illumina kit was not yet available, and the assay components were therefore purchased separately. The study was initiated prior to the Illumina-GenoScreen collaboration through which the combined kit became available, and before the establishment of the Global Health Access Initiative (GHAI). Although kit completeness reduced logistical variability, several technical challenges emerged during library preparation. The workflow remained fully manual, requiring repeated small-volume pipetting and bead-based cleanup steps, thereby increasing operator-dependent variability and limiting throughput, particularly when processing large batches.

Beyond operator-dependent challenges, the composition of clinical specimens also influenced sequencing efficiency. Because tagmentation (the simultaneous fragmentation of DNA and addition of sequencing adapters) is non-selective, any residual host DNA present at the library preparation stage may be fragmented and sequenced, particularly in sputum-derived specimens (19). Although Deeplex Myc-TB is a targeted assay, the total number of sequencing reads generated does not always translate directly into diagnostically informative *M. tuberculosis* target reads (20). Specimen-derived background DNA or other non-informative library material may generate sequencing reads that are not used for diagnostic interpretation in the Deeplex Myc-TB web analysis pipeline, but may reduce sequencing efficiency by consuming reads that would otherwise contribute to effective *M. tuberculosis* target coverage depth. Strategies to increase the proportion of informative *M. tuberculosis* target reads should therefore focus on minimising host DNA and non-specific amplification, for example, through additional cleanup or size selection, host-DNA depletion, or by refining upstream extraction (21, 22). DNA input thresholds also proved critical at this stage of the workflow: while sequencing succeeded with individual library concentrations as low as 0.45 ng/μL, consistent success required maintaining libraries above ~1 ng/μL. While the manufacturer’s protocol specifies ~1 ng total DNA per library as the recommended input, we empirically observed that higher inputs (up to ~100 ng total DNA) were compatible and improved consistency in our workflow, particularly for difficult specimen types such as sputum sediment.

**Figure 1:**
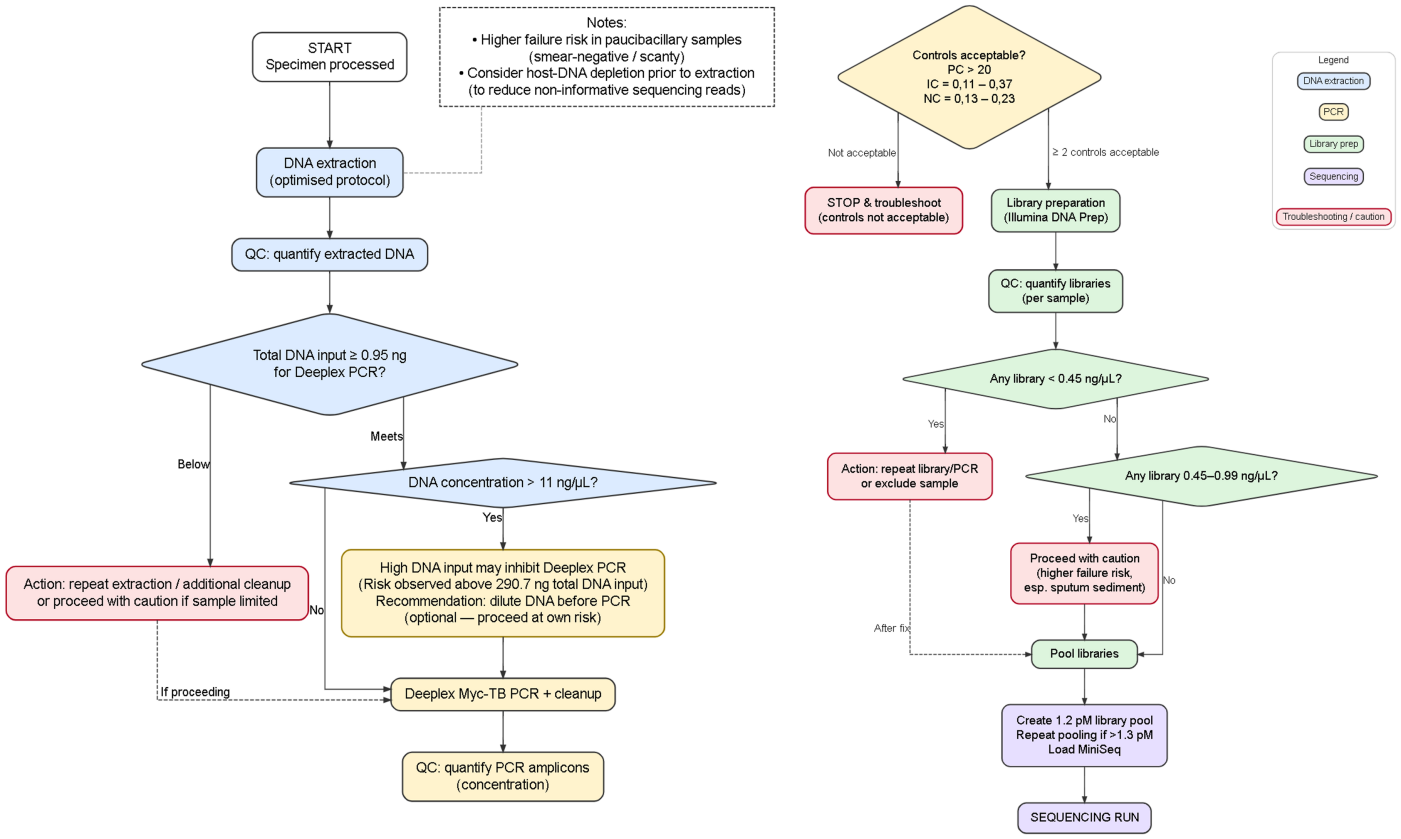
Quality control decision tree used to guide progression through Deeplex Myc-TB DNA extraction, PCR, library preparation, and sequencing. The decision tree summarises the main quality control thresholds used during workflow optimisation, including DNA input, PCR product assessment, control acceptability, library concentration, and MiniSeq loading concentration. Figure was generated in RStudio using R version 4.5.1.

Optimisation of sequencing performance required careful adjustment of library loading concentrations. The Illumina MiniSeq manual recommends a final pooled library concentration of 1.2–1.3 pM (23). Over-clustering, reflected by excessive signal intensity on the flow cell, was observed at 1.3 pM. Standardising the final loading concentration to 1.2 pM across runs was a practical adjustment that improved reproducibility, regardless of variation in individual library concentrations. The quality control thresholds used to guide progression to sequencing are summarised in the decision tree (Figure 1). Workflow performance across 18 sequencing batches is summarised in Supplementary Table 4. Of 330 sample libraries included in sequencing success calculations, 206 achieved at least a “+” quality score in the Deeplex web application. These libraries largely reflected sputum sediment specimens processed during workflow optimisation.

The Illumina MiniSeq platform performed reliably under routine laboratory conditions when reagent integrity, library loading concentration, and DNA input thresholds were well controlled. The compact design and user-friendly interface made it well suited for low- to medium-throughput (≤24 libraries per run). However, sequencing below full cartridge capacity increases per-specimen costs and may necessitate batching of clinical specimens, potentially introducing delays. For routine implementation, tNGS would ideally be placed in a centralised or regional laboratory with sufficient numbers of eligible specimens to support regular full-batch sequencing. Where specimen flow is inconsistent, laboratories would need to balance batching efficiency against clinically acceptable turnaround times.

From a pre-implementation feasibility and sustainability perspective, reliance on platform-specific reagents introduced additional vulnerabilities. Dry-ice shipment requirements substantially increased costs, and repeated freeze–thaw cycles of library preparation reagents can degrade performance, underscoring the importance of consistent reagent handling and proper storage conditions. As an example, the originally recommended Nextera Flex kit was discontinued during the study period. While alternative kits (e.g., Illumina DNA Prep and Nextera XT) are available, they continue to depend on cold-chain transport and are susceptible to supply chain constraints and price fluctuations. This highlights the importance of selecting workflows with resilient supply chains and sustainable procurement pathways when implementing tNGS in LMIC settings. Sustained vendor support, regular software maintenance, and planning for platform transition remain important to further prevent workflow disruption due to hardware failure, delayed technical support, or platform obsolescence, particularly in geographically isolated laboratories. Any transition from MiniSeq to newer Illumina platforms would require workflow review and assay-specific validation before routine implementation.

#### 2.3.4 Data analysis and reporting

The automated Deeplex web application streamlined data analysis and produced comprehensive reports summarising resistance-associated mutations, coverage depth, and lineage information. Its colour-coded format simplified result interpretation, supporting efficient review by users with limited bioinformatics expertise. Data uploaded to the GenoScreen Deeplex Myc-TB platform are processed via Illumina’s secure cloud infrastructure, which provides encrypted transfer, restricted access, and user data ownership in accordance with international data protection standards (24).

Despite these strengths, several technical and operational considerations were identified during routine use. The Deeplex user manual cautions against uploading compressed FASTQ files exceeding 100 MB per read file (R1 or R2) (12), which may fail to analyse on the Deeplex Web Application. When files exceeded this limit, we first attempted decompression, followed by recompression to repack the compressed FASTQ files; when this was unsuccessful, reads were reduced to meet the upload threshold by subsampling and/or filtering for *M. tuberculosis*–aligned reads using genoSubSampler (25). The platform’s pay-per-analysis model also had practical implications for quality assurance: when software patches were released to address changes in heteroresistance calling thresholds and classification behaviour between software versions, reanalysis of previously processed datasets required additional credits, increasing the cost of troubleshooting and result verification. In addition, reliance on internet connectivity and remote processing occasionally delayed analysis turnaround. However, once analysis was initiated, processing continued reliably and did not require restarting if the web session was closed. Development of an offline analysis option [e.g., TB-Profiler (26)] could increase accessibility for laboratories in regions with unreliable internet connectivity; however, further validation would be required to confirm performance on targeted sequencing data.

We also noted challenges related to the resistance catalogue and software versioning. During the study period, the application relied on version 1 of the WHO mutation catalogue, which was already 2 years outdated at the time of initial testing. Given the evolving evidence base for resistance-associated mutations, regular catalogue updates are required to maintain clinical relevance. However, at the time, no automated update pathway was available, and version updates introduced uncertainty around result comparability. For example, the criteria used to grade sequence quality (“sequence acceptability scores”) are not disclosed, and thresholds even changed between software versions (e.g., from “2+” to “1+” when updating from Deeplex Myc-TB V3_0_1, incorporating the 2021 WHO mutation catalogue, to Deeplex Myc-TB V4_0_2). This reduced transparency made it more difficult to interpret borderline results across versions. In this context, borderline results refer to sequencing outputs close to the analysis pipeline’s internal quality and reporting thresholds (e.g., sequence acceptability scores and coverage-based criteria), where differences between software versions could influence result acceptance and/or the reporting of low-frequency (heteroresistant) variants. Uncharacterised or “blue” variants reported by the Deeplex web application represented variants for which resistance or susceptibility could not be assigned without additional supporting evidence. In our context, these variants were not interpreted as resistant or susceptible but were assessed alongside available pDST results and expert review before clinical relevance was considered.

In addition, platform-specific bioinformatic artefacts were observed that affected downstream interpretation. Occasional trimming-related artefacts during FASTQ processing produced false variant calls, including spurious insertions. For example, we encountered a recurring erroneous uncharacterised *rrl* variant misclassified as a heteroresistant mutation present in ~3–11% of sequence reads. Subsequent experiments indicated that this artefact was specific to MiniSeq sequencing (i.e., it was not observed on MiSeq) and was ultimately attributed to trimming behaviour within the analysis pipeline. These findings underscore the importance of independent verification processes and cautious interpretation of unexpected results, particularly when bioinformatic workflows are vendor-managed and local users have limited visibility into intermediate steps.

#### 2.3.5 Procurement, operational considerations, and costs

Close collaboration with regional distributors enabled rapid equipment installation and timely technical assistance during setup. In this study, the local distributor became involved at the start of the experimental work and supported implementation through hands-on training, troubleshooting, and escalation of technical queries to vendors when required, which helped maintain operational continuity during early workflow challenges. Nevertheless, procurement delays remained common because of import requirements, customs clearance, and limited availability of key reagents within South Africa. Illumina sequencing kits required extended lead times, often exceeding 6 weeks, which disrupted scheduling and threatened continuity.

To mitigate these constraints, local procurement frameworks or regional reagent repositories should be established, to the extent possible, to shorten turnaround times and reduce dependence on international suppliers. Formalising service agreements with distributors to specify response times, escalation pathways, and access to technical support and spare parts strengthens long-term workflow reliability. Collectively, these measures should specifically address the sustainability risks posed by prolonged procurement processes and shipping costs, particularly in settings where reliance on a limited number of suppliers increases vulnerability to supply chain disruptions and pricing fluctuations (27).

Although the kit-based format supported consistency through stable reagents and long shelf life, the process remained time-consuming, multi-step, and dependent on precise manual handling. Automation of key manual steps may reduce hands-on time, contamination risk and operator variability, but is costly to procure and maintain. Cold-chain shipment requirements further increased implementation costs. Key operational factors influencing sequencing success and implementation feasibility are summarised in Table 1, while a summary of consumables costs is provided in Supplementary Table 1 and the detailed cost calculations are provided in Supplementary file 2. The estimated total consumables cost per sequencing run was US$ 3797.89 (R 59740.81), corresponding to approximately US$ 179.77 (R 2827.78) per specimen when performed at full run capacity, consistent with prior cost estimates (28, 29). Our ~ 36-h turnaround time (TAT) estimate reflects the minimum technical workflow time under optimal conditions and should not be interpreted as routine clinical turnaround time. During our study, the observed workflow was completed over 4 days after specimen batching, as shown in Supplementary Figure 5.

**Table 1:** Operational factors influencing sequencing success and implementation feasibility.

Domain	Parameter	Manual Deeplex Myc-TB workflow	Lessons learnt
Throughput	Practical batch size	Up to ~24 (21 specimens and 3 controls) libraries/run (MiniSeq), batching often required	Batches below full capacity (<24) increase cost per library and could delay reporting if waiting for full batches
Turnaround time	Total TAT	Minimum hands-on/technical workflow time: ~ 36 h under optimal conditions. Observed practical workflow: 4 days after specimen batching (Supplementary Figure 5)	Sequencing runtime is fixed. Optimisation affects delays prior to and after sequencing
Laboratory workload	Hands-on time	High (multi-step manual pipetting/cleanup)	Manual handling increases variability and error risk
Input challenges	Paucibacillary specimens	Lower success probability	Low MTB DNA yield and inhibitors reduce amplification efficiency and amplified product yield
QC decision points	Minimum acceptance criteria	Controls ≥2/3 pass; library ≥1 ng/μL preferred	Clearly defined quality parameters support decision-making and prevent expensive failed runs
Sequencing setup	Loading concentration	1.2 pM reliable; >1.3 pM risks over-clustering	Minor deviations affect run quality, e.g., over-clustering
Data analysis	Pipeline model	Credit-based and cloud dependent	Reanalysis costs and internet dependence can affect continuity
Supply chain	Reagent availability	Cold chain shipping and long lead times	Procurement delays disrupt continuity
Sustainability	Vendor support pathway	Escalation required	Response time and transparency matter for troubleshooting
Cost	Kits/consumables cost per run	US$ 179.77 (R 2827.78) per specimen (at full capacity), US$ 3797.89 (R 59740.81) per run (Supplementary Table 1; detailed calculations in Supplementary File 2)	Affordability depends on throughput and failures avoided

## 3 Discussion

This study did not represent full routine implementation of the Deeplex Myc-TB assay with real-time patient testing in parallel to standard diagnostic care. Instead, it provided a structured evaluation of the workflow requirements for future implementation of the Deeplex Myc-TB assay in a high-burden area in South Africa. Across the workflow, the findings showed that the assay’s potential value is closely linked to the quality of input material, the addition of appropriate quality control steps, and the availability of trained staff, dependable reagent supply, and sustainable data analysis support.

Several lessons are relevant for laboratories considering similar work and we summarise the key considerations in Table 1. First, workflow implementation readiness cannot be judged on kit design alone; specimen type, specimen-associated inhibitory effects, and extraction performance remain decisive. Second, manual workflows can be established in well-resourced laboratories, but they introduce operator-dependent variability, create throughput constraints, and require greater technical expertise and more intensive hands-on training for staff. Third, proprietary web-based analysis and platform-specific reagents can simplify adoption but also create dependencies that affect transparency and long-term sustainability. Our study-specific adaptations of the manufacturer-recommended workflow are summarised in Supplementary Table 2.

Together, these observations show that implementation planning for tNGS requires attention to multiple linked workflow components, rather than assay performance alone. We summarised the strengths, weaknesses, opportunities and threats to provide a SWOT analysis in Supplementary Figure 4. These findings may help suitably equipped laboratories in high-burden areas design more realistic Deeplex Myc-TB assay workflow roadmaps while avoiding overestimation of immediate routine readiness.

## 4 Conclusion

This study demonstrated that careful planning and consideration are required when implementing a tNGS strategy such as Deeplex Myc-TB. Our findings suggest that Deeplex Myc-TB is technically implementable in a suitably equipped laboratory, but routine diagnostic implementation remains conditional on sustainable infrastructure, supply chains, batching capacity, quality control, and validated platform options.

## Data Availability

The original contributions presented in the study are included in the article/Supplementary material, further inquiries can be directed to the corresponding author.
