# Supplementary material for: Evaluation of the GenoScreen Deeplex Myc-TB workflow for drug-resistant tuberculosis testing in South Africa
Source: Front Public Health. 2026 Jul 7;14:1834699. doi: 10.3389/fpubh.2026.1834699 (PMC13385177; doi:10.3389/fpubh.2026.1834699)
Supplement: Supplementary file 1 [file Supplementary_file_1.DOCX]

**Evaluation setting and intended use**

The Deeplex Myc-TB assay was evaluated at the NHLS, Green Point, Cape Town, South Africa. This centralised facility serves as the primary reference laboratory for the Western Cape Province, and processes approximately 40,000 to 60,000 TB-related diagnostic tests monthly from 53 hospitals and 170 primary healthcare facilities. The laboratory performs routine molecular diagnostics and pDST for clinical isolates across the province. Although equipped for conventional PCR-based diagnostics, the laboratory was not originally designed for sequencing workflows. To support the Deeplex Myc-TB evaluation, we collaboratively implemented procedural controls to minimise contamination risk, including separation of pre- and post-amplification activities.

Patients provided two sputum specimens, the first of which was used for *Mycobacterium tuberculosis* (MTB) detection and rifampicin resistance testing via the Xpert MTB/RIF Ultra assay, BD MAX MDR-TB or the Roche cobas MTB-RIF/INH. If MTB and rifampicin resistance was detected, a second specimen was collected and underwent NALC-NaOH decontamination, centrifugation, and resuspension of the sediment in phosphate-buffered saline. This sediment was divided for downstream procedures, including liquid culture in mycobacterial growth indicator tubes (MGIT) for pDST, extended molecular testing with the Xpert MTB/XDR assay, and smear microscopy. The NHLS Green Point laboratory diagnoses an estimated 18 to 20 new rifampicin-resistant TB (RR-TB) cases each week. To integrate tNGS into this workflow, aliquots of the remaining sputum sediment or MGIT cultures were processed for DNA extraction and sequencing. This design allowed sequencing to proceed in parallel with routine diagnostic processes, maintaining continuity of care and minimising disruptions to standard laboratory operations.

Integrating tNGS alongside existing diagnostic workflows required coordination to avoid disrupting routine clinical services. Aliquots from MGIT cultures or decontaminated sputum sediments were used for sequencing (**Supplementary Figure 1**). Aliquoted MGIT cultures were divided into two portions, with one archived and the other heat-inactivated prior to processing. Decontaminated sputum sediments (≥1 mL) were similarly heat-inactivated before DNA extraction. As these specimens were considered biohazardous/infectious prior to heat-inactivation, all pre-inactivation handling steps occurred in a biosafety level 3 (BSL-3) facility. All tNGS data processing was conducted independently of routine diagnostics, and results were excluded from clinical decision-making. This model allowed tNGS to operate as an overlay on routine diagnostics rather than a replacement, without disrupting clinical reporting timelines. It also enabled evaluation of a partially integrated workflow for future implementation within the national TB diagnostic network. DNA extraction, amplification, and sequencing followed a weekly batching schedule, enabling consistent throughput while maintaining manageable workloads for the laboratory staff.

- 1. **Preparation phase**

Three rooms were designated as amplicon-free or post-amplification areas to maintain a unidirectional workflow, with separate zones for reagent preparation, DNA extraction, amplification, and library preparation (**Supplementary Figure 2**). The Illumina MiniSeq sequencer (Illumina, Inc, San Diego, California) was installed in an existing temperature-controlled molecular diagnostics laboratory, leveraging an already controlled environment. Additional instruments included a Qubit 4 fluorometer (Thermo Fisher Scientific, Massachusetts, United States), dead-air boxes, centrifuges, vortex mixers, and dedicated pipettes for pre- and post-PCR activities. A compact deep freezer was also installed for bulk reagent storage.

To address intermittent power interruptions, an uninterruptible power supply (UPS) was installed in the post-amplification area to maintain uninterrupted operation and protect the cold chain. We also established local storage and archiving for sequencing outputs and reports, as downloadable Deeplex reports are only retained for one year. Together, these upgrades enabled sustainable sequencing within a high-throughput diagnostic setting.

**Regulatory authorisation:** In addition to the ethical approvals provided in the main text, authorisation for the importation of an unregistered medical device was granted by the South African Health Products Regulatory Authority (SAHPRA, Section 21 authorisation MD20240702), in terms of the Medicines and Related Substances Control Act, 1965 (Act 101 of 1965), to permit importation of the assay kit for evaluation on clinical specimens in this setting.

**1.3 Capacity development, training, and workflow discipline**

Laboratory personnel began formal training in November 2022 through eight structured sessions with application specialists, followed by hands-on practice and supervised sessions as the workflow was established through August 2023.

Trainees practiced the workflow from DNA extraction to sequencing output, including quality control procedures such as DNA quantification and library visualisation using the 4150 TapeStation system (Agilent Technologies, California, United States). Initial formal training equipped laboratory personnel with the skills to perform the workflow; however, early sequencing runs encountered several failures, underscoring the need for continued supervision and iterative retraining. Following each failed run, workflow steps were repeated under direct supervision to identify operator-dependent errors and opportunities for workflow improvement, as described below.

Training also emphasised workflow discipline. Technicians were required to adhere strictly to unidirectional movement through laboratory spaces, ensuring that personnel did not re-enter DNA extraction areas after exposure to amplicons (**Supplementary Figure 2**). Additional training covered contamination prevention, data handling, sequencing instrument operation, and adherence to good laboratory practice (GLP).

1. **Supplementary figures**


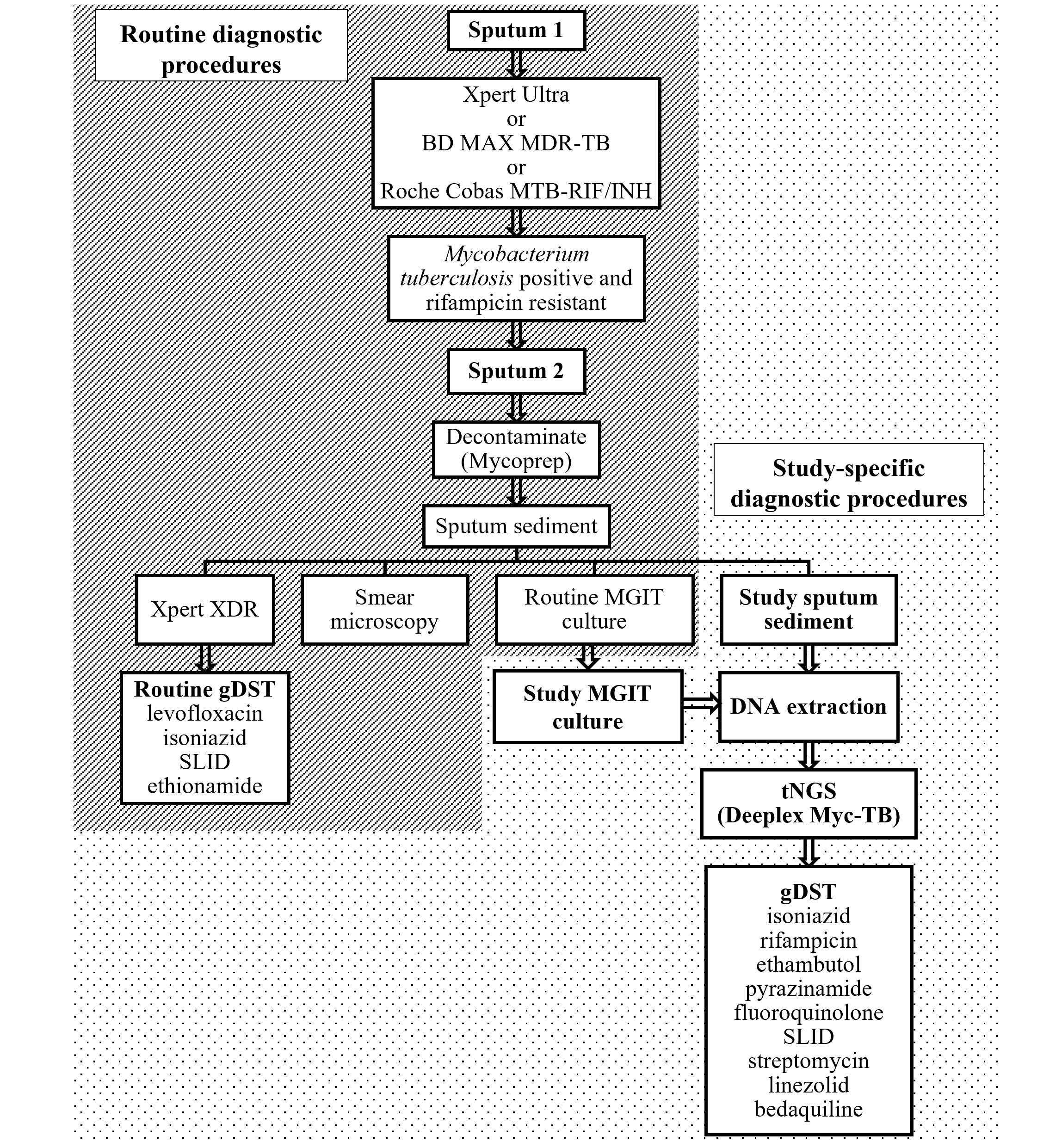


**Supplementary Figure 1. Integration of study-specific and routine diagnostic procedures.** Overview of specimen and workflow integration between standard-of-care (routine) and study-specific procedures conducted at NHLS Green Point.

*gDST: genomic drug susceptibility testing.

*Collected only when sputum 1 tested MTB-positive and rifampicin-resistant on the initial assay. Specimens without detected rifampicin resistance would not enter this diagnostic pathway. As a result, rifampicin resistance missed by the initial assay, or isoniazid mono-resistance in the absence of rifampicin resistance, may not be captured in this context.


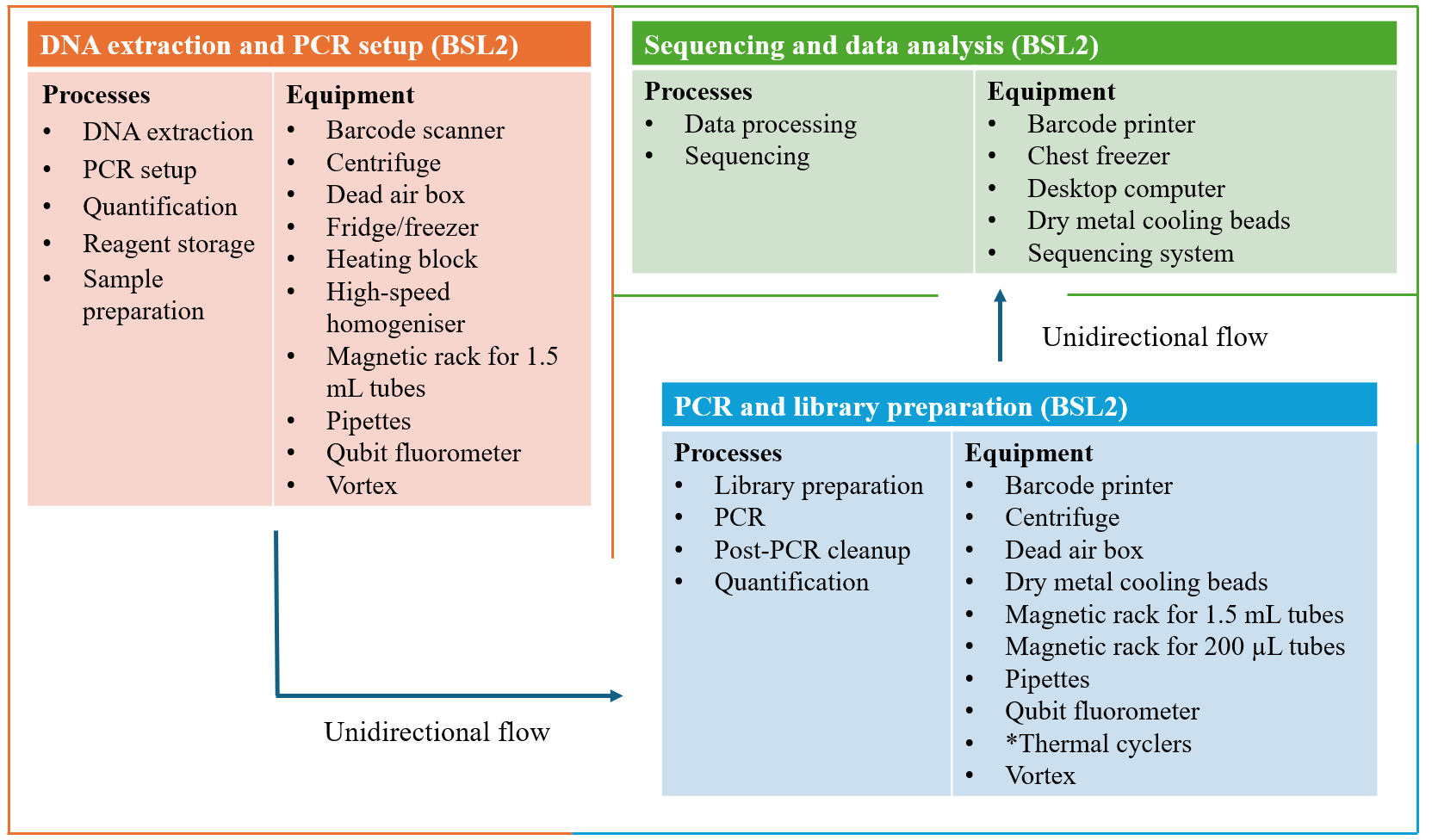


**Supplementary Figure 2.** Floor plan of the laboratory showing the unidirectional Deeplex Myc-TB workflow, room functions, equipment placement, and colour-coded workflow zones. Staff and specimens moved together in one direction, with no return from post-amplification to pre-amplification rooms. Standard laboratory personal protective equipment (PPE) included lab coats, gloves, closed shoes, and tied-back hair where applicable.

Orange: DNA extraction and PCR setup (BSL2)

Blue: PCR and library preparation (BSL2)

Green: Sequencing and data analysis (BSL2)

* NHLS-provided equipment


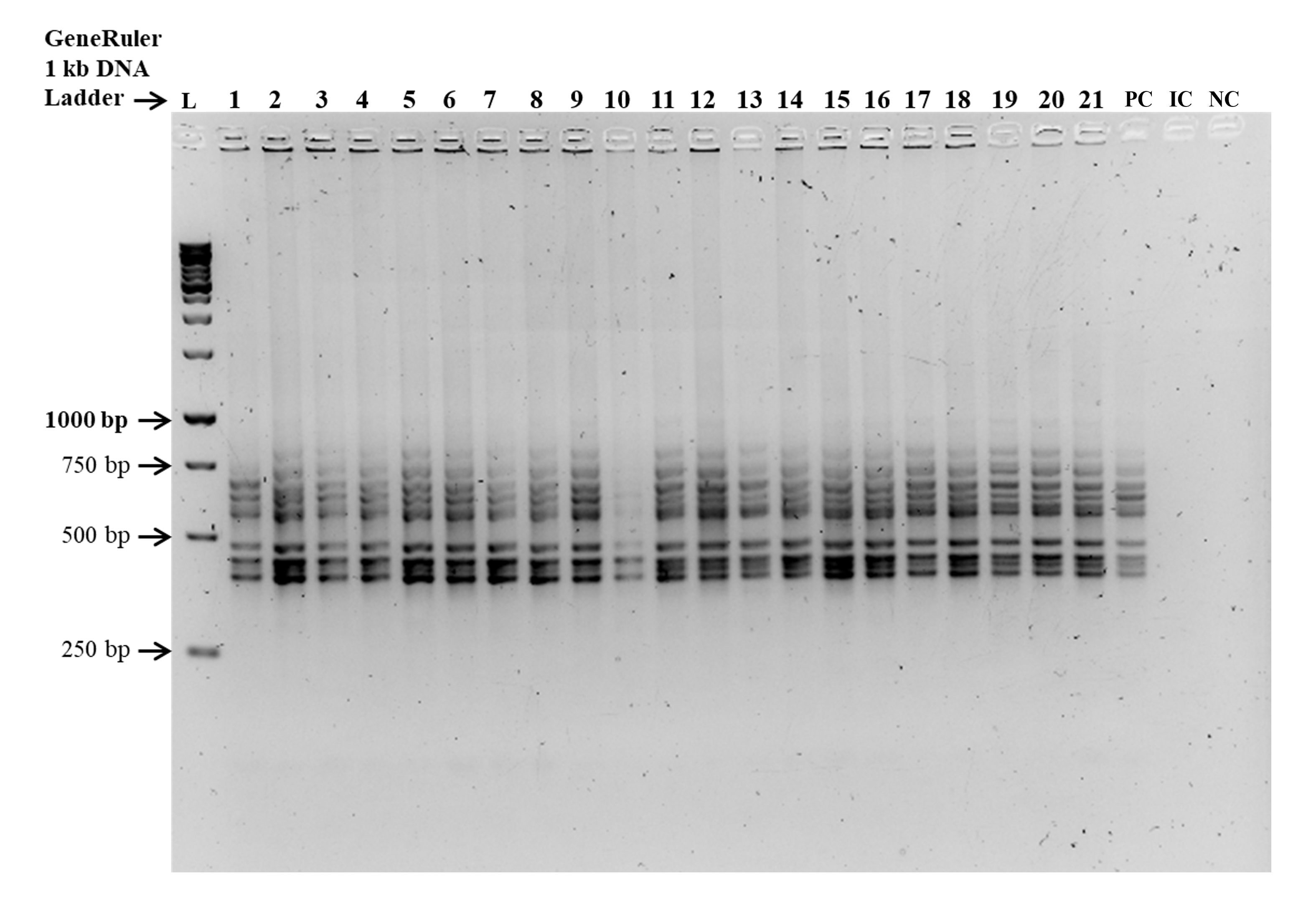


**Supplementary Figure 3. Gel electrophoresis of Deeplex Myc-TB assay-derived amplified products.** A 2% agarose gel was run at 100 V for one hour to visualise post-PCR cleanup amplicons from all reactions, including controls. We used the positive control lane as a reference for PCR efficiency on the gel images. From left to right, lane 1: (L) GeneRuler 1 kb DNA Ladder (Thermo Fisher Scientific, Waltham, MA, USA), lanes 2 – 22: individual amplified products from reactions 1 – 21, lanes 23 – 25: manufacturer-recommended PCR quality control, including a positive control (PC), internal control (IC), and negative control (NC). Gel electrophoresis was used during the optimisation phase as an additional quality control (QC) tool but was later discontinued as a routine QC step because weak or absent bands were not always predictive of downstream sequencing failure. As familiarity with the workflow increased, earlier QC indicators and observed sequencing success patterns were used to guide decisions more reliably.


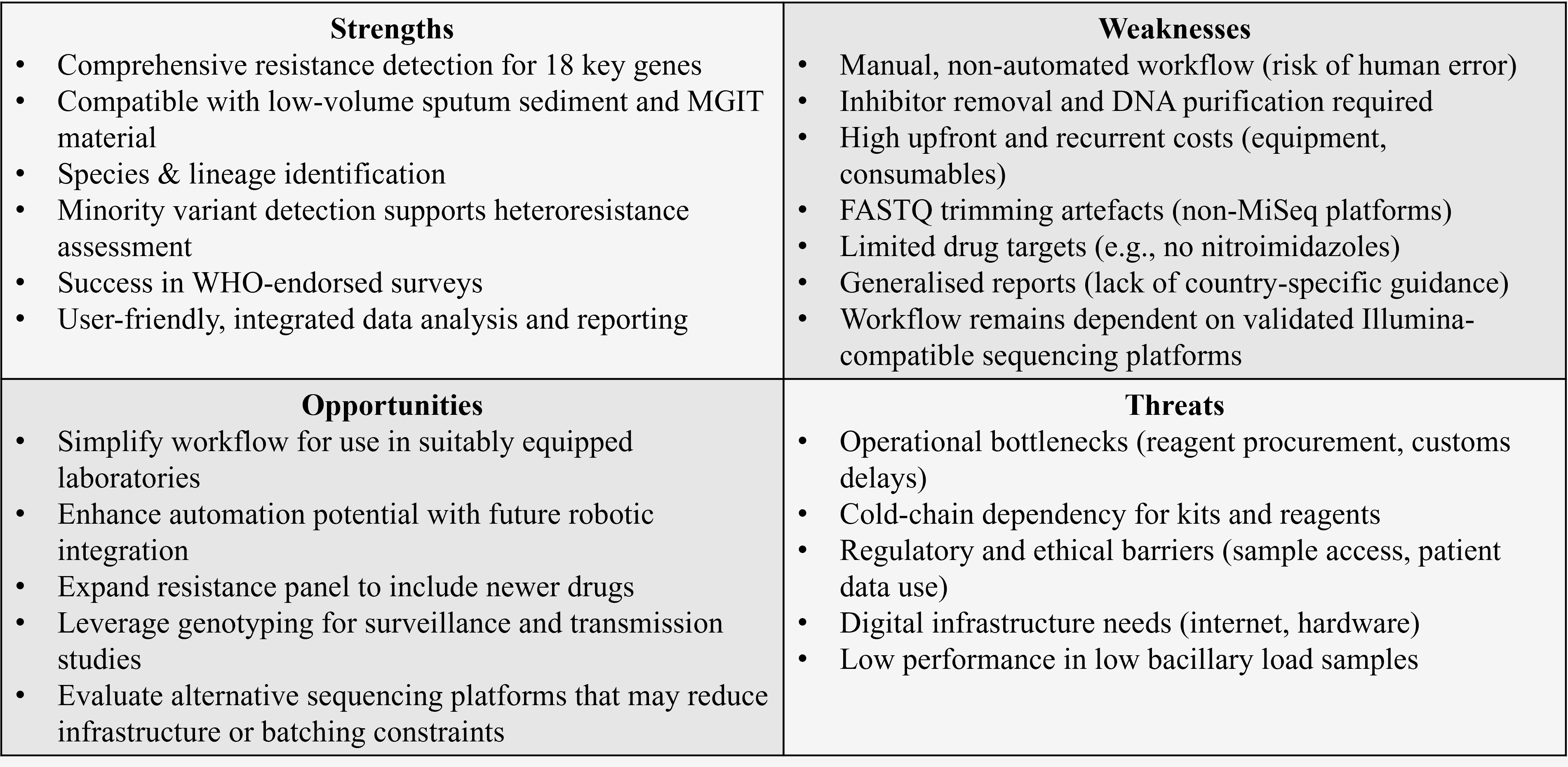


**Supplementary Figure 4: SWOT analysis of the Deeplex Myc-TB assay workflow and considerations for future implementation.** A visual summary of the strengths, weaknesses, opportunities and threats identified during evaluation of the Deeplex Myc-TB workflow in this study.


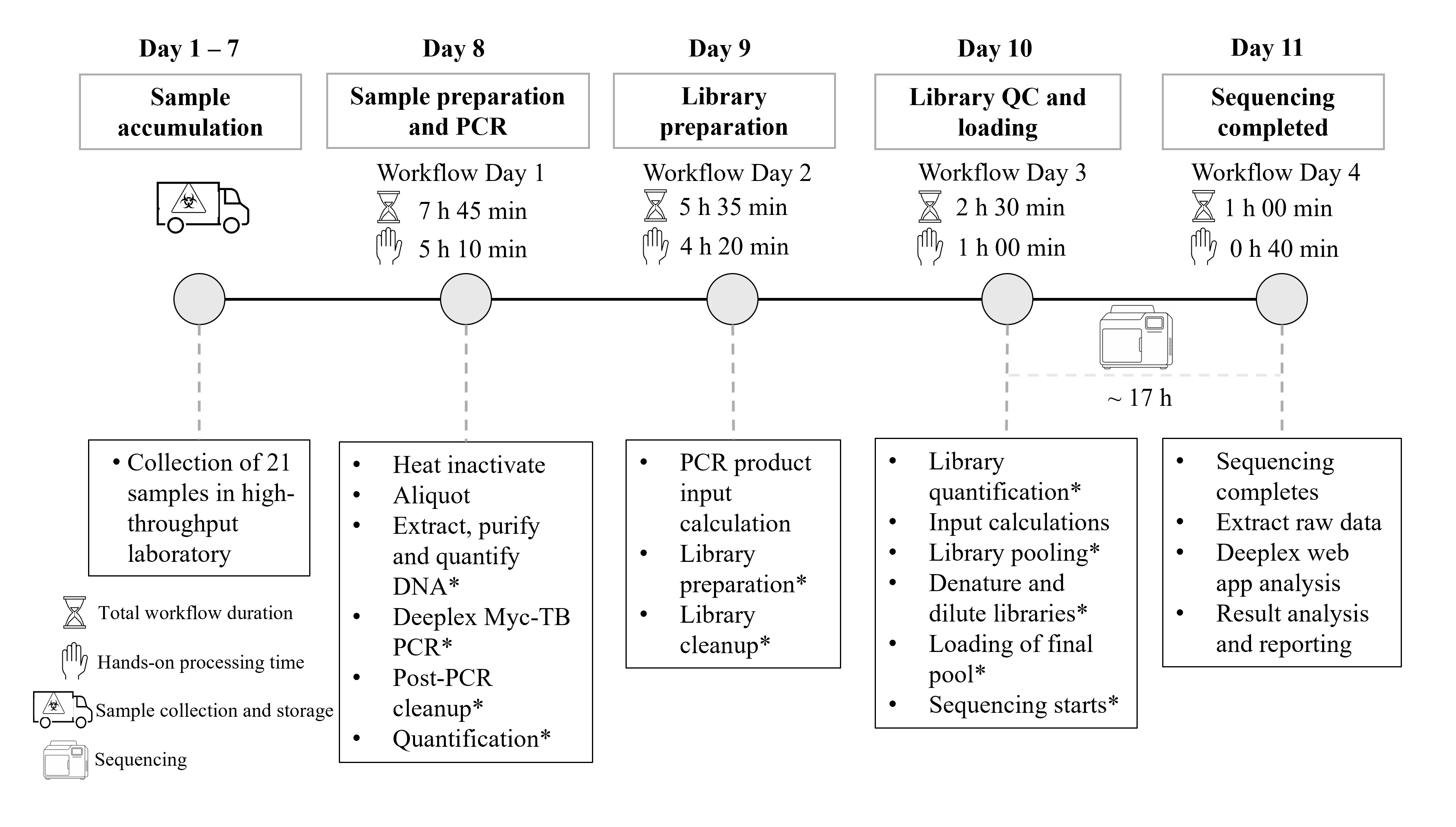


**Supplementary Figure 5:** **Observed Deeplex Myc-TB assay workflow turnaround time under well-planned/resourced conditions.** Workflow duration includes hands-on processing and in-process waiting steps. Hands-on time refers to active operator time. Estimates are based on 8-hour workdays for a 21-specimen batch with three controls. The timed workflow stages shown in the figure sum to ~ 34 h. The reported ~ 36 h TAT also includes routine operational handling that is not itemised separately, including workspace preparation and decontamination after use, PPE changes, movement between designated laboratory areas, and reagent and equipment preparation. Cartridge thawing/preparation before sequencing is shown as an in-process waiting step and is performed after library quality control confirms successful library preparation. Specimen accumulation time is excluded from the minimum technical workflow duration and depends on specimen flow. In this example, batching is based on high-throughput conditions where at least 21 rifampicin-resistant TB cases are identified per week.

QC, quality control.

1. **Supplementary tables**

**Supplementary Table 1.** Summary of consumables costs for Deeplex Myc-TB tNGS on clinical specimens.

| **Category** | **Workflow/Item** | **Consumables/kits cost per run, USD (ZAR, VAT excl.)** | **Cost per clinical specimen, USD (ZAR, VAT excl.)** |
| --- | --- | --- | --- |
| **Wet-lab workflow (consumables)** | Aliquot specimens + DNA extraction + purification *(21 specimens only)* | **44.49 (699.83)** | **2.05 (32.25)** |
|  | Deeplex PCR + post-PCR cleanup *(21+3 controls; QC included)* | **53.25 (837.62)** | **2.67 (42.00)** |
|  | Library prep + cleanup + pooling *(21+3 controls; QC included)* | **108.41 (1705.29)** | **9.25 (145.50)** |
|  | QC only (Qubit dsDNA HS) *(21+3 controls)* | **20.95 (329.54)** | **1.03 (16.20)** |
|  | **Subtotal: Wet-lab consumables** | **227.10 (3572.28)** | **15.00 (235.95)** |
| **Sequencing reagents/kits** | Illumina + GenoScreen combo kit *(48 libraries; 24 libraries/run)* | **48 libraries: 4711.95 (74118.97)**  **24 libraries: 2662.43 (41880.02)** | **126.78 (1994.25)** |
|  | MiniSeq mid output kit (cartridge, flow cell and HT1 buffer) (*24 libraries)* | **883.49 (13897.30)** | **36.81 (579.02)** |
|  | PhiX control v3 *(fixed per run)* | **24.87 (391.21)** | **1.18 (18.56)** |
|  | **Subtotal: Sequencing reagents/kits (combo model)** | **3570.79 (56168.53)** | **164.77 (2591.83)** |
| **TOTAL** | **Grand total per run (wet-lab + sequencing reagents)** | **3797.89 (59740.81)** | **179.77 (2827.78)** |

**Note:** Costs are presented in USD, with ZAR values in parentheses, excluding VAT. USD equivalents were calculated using an exchange rate of US$1 = ZAR 15.73 (29 January 2026).
* Estimates include consumables, reagents, and kits only; staff time, equipment, service/maintenance, and overhead costs are excluded.

*Per-specimen costs assume 21 clinical specimens and 3 controls per sequencing run and increase when fewer libraries are sequenced because cartridge/flow cell capacity is fixed at 24 libraries.

*Prices may vary by supplier and over time.
**Abbreviations**: ZAR, South African rand; VAT excl., value-added tax excluded.

**Supplementary Table 2.** Summary of study-specific adaptations of the manufacturer-recommended steps within the Deeplex Myc-TB assay workflow.

| **Manufacturer-recommended step** | **Study-specific adaptation** | **Rationale** |
| --- | --- | --- |
| Manufacturer-recommended manual DNA extraction method (bead-beating and ethanol precipitation-based) | A modified InstaGene/FastPrep extraction method with AMPure XP bead purification was used | The manufacturer-recommended extraction approach did not consistently produce DNA of sufficient quantity and purity under local laboratory conditions. The modified method improved DNA quality and consistency while remaining feasible in a resource-limited area. |
| Reconstitution of the Deeplex internal control with 897 µL nuclease-free water | Internal control reconstituted with 448.5 µL nuclease-free water | This modification was introduced during troubleshooting after repeated internal control failures within the same kit lot. The reduced volume was intended to improve dissolution and support more consistent control performance. |
| Internal control reconstitution according to the manual, with no heating step specified | Internal control reconstituted using nuclease-free water preheated to 90 °C | Preheating was introduced to promote more complete dissolution of the control material and reduce the risk of false internal control failure due to incomplete resuspension. |
| Amplification assessment based primarily on post-cleanup amplicon quantification thresholds | Gel electrophoresis added as an additional quality control step before sequencing during workflow optimisation | Qubit-based quantification alone did not reliably confirm multiplex completeness or the presence of all expected amplicons. Gel electrophoresis provided a simple visual confirmation of amplification success before library preparation. |
| Approximate PCR input of 900 ng total DNA | DNA extracts with substantially lower input were retained in the workflow, with successful amplification observed at 0.95 ng total DNA input | Study findings showed that amplification could still succeed well below the nominal recommended input, allowing lower-yield specimens to remain eligible for testing. |
| Recommended library input of approximately 1 ng total DNA | Higher library inputs, up to approximately 100 ng total DNA, were accepted | Higher library inputs were compatible with sequencing in this setting and improved consistency, particularly for more challenging specimen types. |
| MiniSeq loading concentration guidance of 1.2 – 1.3 pM. 1.5 pM was used during initial vendor-supported training runs | Final pooled library loading concentration standardised to 1.2 pM | Initial vendor-supported training runs used 1.5 pM, while subsequent testing showed over-clustering at 1.3 pM. Standardisation to 1.2 pM improved sequencing data output quality. |
| Deeplex Web Application upload limit of 100 MB per compressed FASTQ read file | Oversized files were recompressed or reduced by subsampling and/or filtering for *Mycobacterium tuberculosis*-aligned reads using genoSubSampler | These steps were used as a practical workaround to meet upload constraints and allow downstream analysis to proceed. |
